# Novel portable hypothermic machine perfusion preservation device enhances cardiac viability of donated human hearts

**DOI:** 10.3389/fcvm.2024.1376101

**Published:** 2024-04-02

**Authors:** Kristina Andrijauskaite, Rafael J. Veraza, Riley P. Lopez, Zach Maxwell, Isabella Cano, Exal E. Cisneros, Israel J. Jessop, Maria Basurto, George Lamberson, Michelle D. Watt, Joseph Nespral, Masahiro Ono, Leonid Bunegin

**Affiliations:** ^1^ Vascular Perfusion Solutions, Inc., San Antonio, TX, United States; ^2^ Texas Organ Sharing Alliance (TOSA), San Antonio, TX, United States; ^3^ Division of Cardiovascular and Thoracic Surgery, Department of Surgery and Perioperative Care, Austin Dell Medical School, University of Texas, Austin, TX, United States

**Keywords:** organ preservation, hypothermic machine perfusion, cardiac grafts, heart transplant, VP.S ENCORE® preservation device, deceased DBD donors, prolonged preservation

## Abstract

**Introduction:**

Heart transplant remains the gold standard treatment for patients with advanced heart failure. However, the list of patients waiting for a heart transplant continues to increase. We have developed a portable hypothermic oxygenated machine perfusion device, the VP.S ENCORE®, to extend the allowable preservation time. The purpose of this study was to test the efficacy of the VP.S. ENCORE® using deceased donors derived hearts.

**Methods:**

Hearts from brain-dead donors not utilized for transplant (*n* = 11) were offered for research from the Texas Organ Sharing Alliance (TOSA), South and Central Texas' Organ Procurement Organization (OPO) and were preserved in the VP.S ENCORE® for 4 (*n* = 2), 6 (*n* = 3), and 8 (*n* = 3) hours or were kept in static cold storage (SCS) (*n* = 3). After preservation, the hearts were placed in an isolated heart Langendorff model for reperfusion and evaluated for cardiac function.

**Results:**

The mean donor age was 37.82 ± 12.67 with the youngest donor being 19 and the oldest donor being 58 years old. SCS hearts mean weight gain (%) was −1.4 ± 2.77, while perfused at 4 h was 5.6 ± 6.04, perfused at 6 h 2.1 ± 6.04, and 8 h was 7.2 ± 10.76. Venous and arterial lactate concentrations were less than 2.0 mmol/L across all perfused hearts. Left ventricular contractility (+dPdT, mmHg/s) for 4 h (1,214 ± 1,064), 6 (1,565 ± 141.3), and 8 h (1,331 ± 403.6) were within the range of healthy human heart function. Thus, not significant as compared to the SCS group (1,597 ± 342.2). However, the left ventricular relaxation (mmHg/s) was significant in 6-hour perfused heart (*p* < 0.05) as compared to SCS. Gene expression analysis of inflammation markers (IL-6, IL-1β) showed no significant differences between SCS and perfused hearts, but a 6-hour perfusion led to a downregulated expression of these markers.

**Discussion:**

The results demonstrate that the VP.S ENCORE® device enhances cardiac viability and exhibits comparable cardiac function to a healthy heart. The implications of these findings suggest that the VP.S ENCORE® could introduce a new paradigm in the field of organ preservation, especially for marginal hearts.

## Introduction

Heart transplants (HT) remain the gold standard treatment for patients with advanced heart disease (1). However, worldwide, there are simply not enough heart donors available to meet the demand (2). In the United States alone, about 20% of patients on the heart transplant waiting list die or become too sick to remain good transplant candidates (3). Approaches to decrease organ shortage include the use of extended-criteria donors (ECDs) or donation after circulatory death (DCD) as well as the emergence of ex-situ machine perfusion (MP) systems as an alternative to the standard of care, the static cold storage (4). It has been estimated that DCD heart transplantation can increase the heart donor pool by about 30% (5). According to the United Network for Organ Sharing (6), 2022 was a record-setting year with a significant increase in DCD heart transplants in the United States. In DCD transplantation, only two techniques can be used: direct procurement and ex-situ perfusion (DPP) using TransMedics OCS, the only approved DCD technology in the United States, and the normothermic regional *in situ* perfusion (NRP) (7). The latter has emerged as a cost-effective alternative with promising early patient outcomes and high organ recovery rates (8, 9). After blood flow restoration, normothermic regional perfusion is followed by the SCS or MP (8). There are two types of machine perfusion technologies: normothermic (NMP) and hypothermic (HMP). Although both of them have their advantages and disadvantages, nevertheless, hypothermic perfusion is deemed to have a safer profile in the event of system failure, as opposed to normothermic machine perfusion (NMP), which carries the risk of causing irreversible damage to the heart (10). While in hypothermic preservation, the organ is cooled to a more standard static cold storage (SCS) temperature ranging from 5 to 10°C, a range considered by the International Society for Heart and Lung Transplantation (ISHLT) as an optimal temperature for the heart (11). Further, NMP involves additional surgical and technical support and appropriate transport, inevitably resulting in a more expensive management cost. While HMP holds tremendous potential for cardiac transplantation, most perfusion devices are quite complex and expensive, requiring blood-based products to be added to the proprietary perfusion solutions. Here, we present data on the HMP of human donor-derived hearts using the VP.S ENCORE®, an innovative and simple-to-use cardiac preservation device, which shows promising preclinical results for prolonged cardiac graft preservation.

## Methods

### Human donor heart procurement

Heart retrieval was performed in a standard fashion through a median sternotomy. The heart was exposed, and the donor was systemically heparinized with 30,000 units of heparin. After sufficient time for heparin circulation, the aorta was cross-clamped and one liter of cold cardioplegia was administered to arrest the heart. The heart was decompressed through the inferior vena cava and left atrial appendage. The donor cardiectomy was completed, and the graft was immersed in a cold saline solution for preparation and inspection.

### Ex vivo hypothermic machine perfusion (HMP) and static cold storage preservation

**Figure 1:**
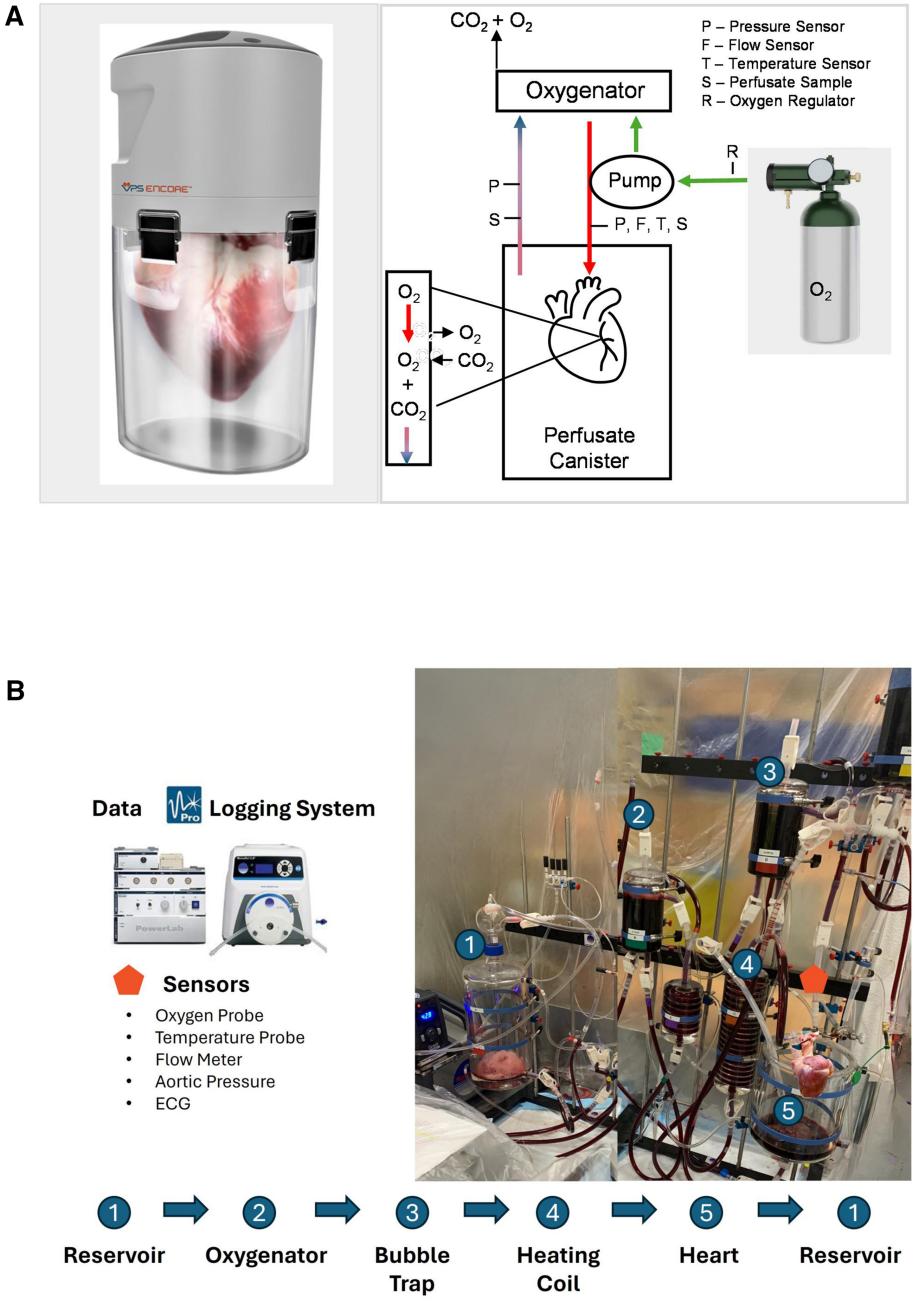
Schematic illustration of the (**A**) VP.S ENCORE® device. An external oxygen tank provides oxygen by pulsing at 50 pulses/min via a micro-solenoid at a fluid driving pressure of 10–20 mmHg. Oxygen travels both to the pump to drive fluid through the system and into the oxygenator to provide oxygen to the solution. The diaphragm pump directs oxygenated fluid directly to the aortic root. The perfusate provides oxygen and nutrients to the heart and fluid exits through the coronary sinus. The pressure gradient drives fluid from the canister and back into the head for reoxygenation. Deoxygenated fluid flows into the oxygenator and carbon dioxide and excess oxygen are vented into the atmosphere. This cycle continues throughout the entire preservation time. Perfusate samples are taken from a cannulated coronary sinus and from the arterial line. The arterial line has sensors to measure pressure, flow, and temperature. A second pressure sensor measures the pressure in the canister, and (**B**) schematic representation of the reperfusion on the Langendorff system where the numbers indicate the major components. To initiate a process, a flush solution consisting of 3l of modified Krebs buffer is circulated through the system using a peristaltic pump. This solution traverses through an oxygenator and bubble trap before entering the aorta. Subsequently, the flush solution is directed to waste. Once the temperature reaches 30°C, the solution is then switched to the main solution containing HBOC. As the heart gradually warms to 39°C, the perfusion flow rate is adjusted accordingly to meet the escalating oxygen demands, facilitating the process of stabilization. Different sensors are instrumented to collect reperfusion parameters, and the data has been continuously recorded using the Data Logging System.

Human donated hearts were either preserved using the VP.S ENCORE® or were kept in static cold storage. After Plasma-Lyte A (1 L) flush, donor hearts in the HMP group were cannulated with aortic and superior vena cava cannula to collect perfusate samples from both arterial and venous ports via oxygen probes. The heart was lowered into the organ storage canister filled with cold (4°C) Belzer MPS® UW Machine Perfusion solution and the perfusion module was secured to the canister with clamps for a liquid-tight seal. The prepared device was lowered into a temperature-regulated vessel and maintained at a hypothermic temperature. The VP.S ENCORE® device is lightweight, portable, and simple to use. The technology is based on hypothermic oxygenated machine perfusion that combines electro-fluidics and mechano-elastic principles to recover the energy inherently stored in compressed oxygen to drive preservation fluid through the coronary arteries of hearts. Compressed oxygen supplies an oxygenator while simultaneously driving fluid through the system into the aorta utilizing a novel diaphragm pump which allows the compact nature of the device and eliminates the need to use roller or centrifugal pumps (Figure 1). During HMP, perfusion flow, pressure, and temperature were recorded at a two-hour interval. Hearts in the static cold storage group were submerged in 1 L of Belzer UW® Cold Storage Solution double-bagged in ice slush and placed in an ice cooler for around 4 h. The perfusate temperature, flow, and pressure were monitored continuously.

### Metabolic and cardiac edema assessment

During HPM, perfusate samples were collected from arterial and venous ports 30 min post the start of perfusion and every two hours afterward. Perfusate samples were analyzed for different blood gases using an i-STAT 200 analyzer (Abbott Laboratories) and cartridges for CG8 (general blood gases), CG4 (lactate), creatinine kinase-muscle/brain (CK-MB) and cardiac TroponinI (cTnI). Lactate expression was measured from both arterial and venous samples, whereas CK-MB and cTnI expression was obtained from only venous samples. The difference in lactate expression was calculated by subtracting arterial values from venous. Cardiac edema was determined by measuring the weights of hearts prior to and after each preservation method.

### Oxygen consumption during perfusion and reperfusion

Myocardial oxygen consumption (MVO2 mlO2/min/100 g) was measured during perfusion and reperfusion. In the latter phase, oxygen consumption was measured throughout an initial 30 min of the rewarming stage (before defibrillation) and after defibrillation. Two oxygen probes (Pyro FireSting™) collected oxygen partial pressure (mmHg) data of the solution before entering the coronary arteries (arterial) and after exiting the cannulated pulmonary artery (venous), whereas flow was measured using the Sensirion flow meter. Oxygen consumption was captured using the following formula, (([O2] a- [O2] v) * Q)/heart weight) * 100. [O2] a and [O2] v are defined as the oxygen content of arterial and venous perfusate respectively. [O2] was calculated as (1.34 * Hb* SO2) + (K * pO2); where 1.34 is ml O2/Hb (g), Hb is the concentration of hemoglobin measured in g/dl, SO2 is the oxygen saturation ratio calculated using the equation developed by Severinghaus (12), where K is the oxygen solubility coefficient adjusted for the perfusate temperature at every data point, pO2 is the partial pressure of oxygen in mmHg for the perfusate sample, and Q is a coronary flow in mL/min.

### Assessment of cardiac function

After preservation, hearts were placed on a Langendorff system with a perfusate mixture of Krebs-Henseleit buffer, PEG-20kD, and hemoglobin-based oxygen carriers (HBOCs). The left ventricular function was expressed as the rate of pressure change over time dP/dT (mmHg/sec) and was measured by placing a pressure catheter (Millar, 5F) in the left ventricle. Data was analyzed using PowerLab (LabChart 8.1.16) blood pressure analyzer. Data was selected from the left ventricular pressure waveform as an average of 30 beats after stabilization (approximately 30 min after defibrillation). Contractility after ionotropic support was measured less than 5 min after administration of epinephrine (0.5 mg injected arterial). The end-diastolic pressure of the left ventricle was maintained by administering perfusate directly into the left ventricle through the mitral valve and venting to the atmosphere.

### Myocardial histological assessment

Tissue biopsies (2.5–4 mm) were obtained pre and post-preservation from left and right ventricle vasculature for histological evaluation. Biopsies were placed in 10% formalin for 24 h and then stored for 3 days in 70% ethanol and embedded in paraffin. Samples were sectioned into 5 μm slides, stained with hematoxylin and eosin (H&E), and were scanned by Amscope microscope-scanner at 20× magnification. Myocardial injury was assessed based on the presence and severity of myofiber necrosis and degeneration, hemorrhage, interstitial edema, endothelial changes, and acute inflammation (13) and it was graded in a semiquantitative scale by an independent, blinded pathologist. Additionally, a subset of the hearts was sent to the cardiac pathology laboratory at the Texas Heart Institute for gross and microscopic evaluation by a blinded certified pathologist.

### Gene expression

Total RNA from cardiac tissue biopsies was extracted following the TRIzol RNA isolation method. The concentration of total RNA was evaluated and measured at 260/280 nm by spectrophotometer (NanoVue Plus, GE Healthcare). Synthesis of cDNA was performed from 0.25 μg of total RNA, which was reverse transcribed using (iScript cDNA Synthesis Kit, Bio-Rad) according to the manufacturer's instructions. Gene-specific pre-designed oligonucleotide primers were purchased from Sigma. qRT-PCR was done using SsoAdvanced Universal SYBR Green Supermix and SFX96 Touch real-time PCR detection system (Bio-Rad, T100 Thermal Cycler). The cycling parameters were as follows: initial denaturation 95°C, 2 min; denaturation 95°C, 5 s; annealing/extension 60°C, 30 s; number of cycles 40; melt curve 65°−95°C (0.5°C increments). The comparative CT (2-ΔΔCT) method was used for all quantification. Values were normalized to the housekeeping gene.

### Statistical analysis

All graphing and statistical analyses were performed using Prism 9 (GraphPad Software Inc., La Jolla, CA, USA). Results were expressed as means ± standard deviation (Std) or means ± standard error of the mean (SEM). Graphs were presented as overall means ± standard deviations/errors. Differences between the groups were assessed using a two-tailed Student's *T*-test for unpaired data. Statistical tests and corrections for multiple comparisons are described in each figure panel.

## Results

### Donor characteristics and reasons for transplant rejection

**Figure 2:**
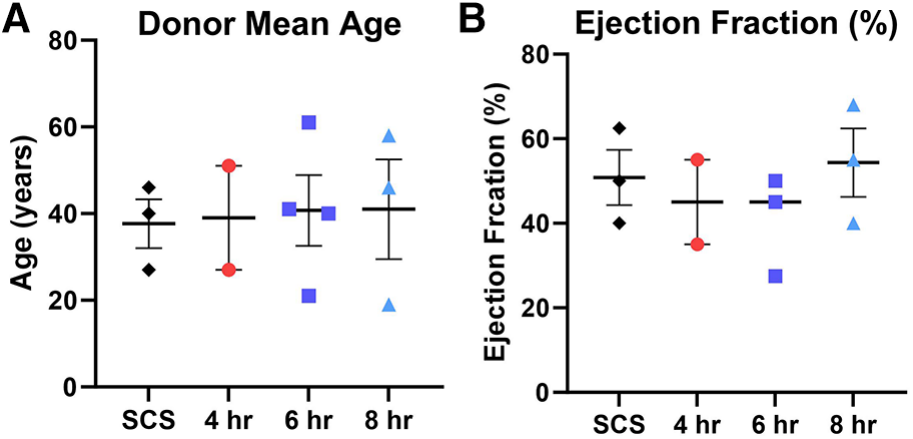
Donor characteristics prior heart procurement based on the preservation group including (**A**) donor mean age, and (**B**) donor ejection fraction (%). Each color represents a different preservation group, while each point represents an individual heart. Values plotted as means ± SEM. Graphs depicting age and ejection fraction of donors exhibit a distribution of data points with indicators for the mean, lowest, and highest values.

To avoid any potential bias, donor hearts were allocated into different preservation groups randomly. Most donor hearts have derived from females (60%) with no significant differences detected among other characteristics prior to heart procurement, such as donor age, ejection fraction, body max index (BMI), and sex (Figure 2). Further, human hearts were not utilized for transplant due to many different reasons, including donor age (*n* = 2), organ size (*n* = 1), rapid recovery (*n* = 1), unacceptable organ-specific test (*n* = 1), organ anatomical defect (*n* = 1), or due to no match run/medical rule out (*n* = 5). Drug overdose was the main cause of death for most donors (*n* = 4), while other causes were accidents, stroke, trauma, sepsis, or unknown reasons (Table 1).

**Table 1:** Donor demographics and reasons for transplant rejection.

Donor characteristics				
Preservation group	SCS (*n* = 3)	4 h (*n* = 2)	6 h (*n* = 3)	8 h (*n* = 3)
Age	37.67 ± 9.71	39.00 ± 16.97	34.00 ± 11.27	41.00 ± 19.97
Ejection fraction (%)	50.83 ± 11.27	45.00 ± 14.14	40.83 ± 11.81	54.33 ± 14.01
BMI	20.70 ± 6.16	23.30 ± 1.28	24.77 ± 3.32	27.23 ± 4.29
Sex (M/F)	2/1	1/1	2/1	0/3
Rejection for transplant				
Donor age		1		1
Organ size				1
Rapid recovery		1		
Unacceptable organ specific test	1			
Organ anatomical defect	1			
No match run/medical rule out	1		3	1
Cause of death				
Drug intoxication	1	1	2	
Accidents				1
Stroke	2		1	
Trauma		1		
Sepsis				1
Uknown				1

### Preservation parameters during HMP and static cold storage (SCS)

**Figure 3:**
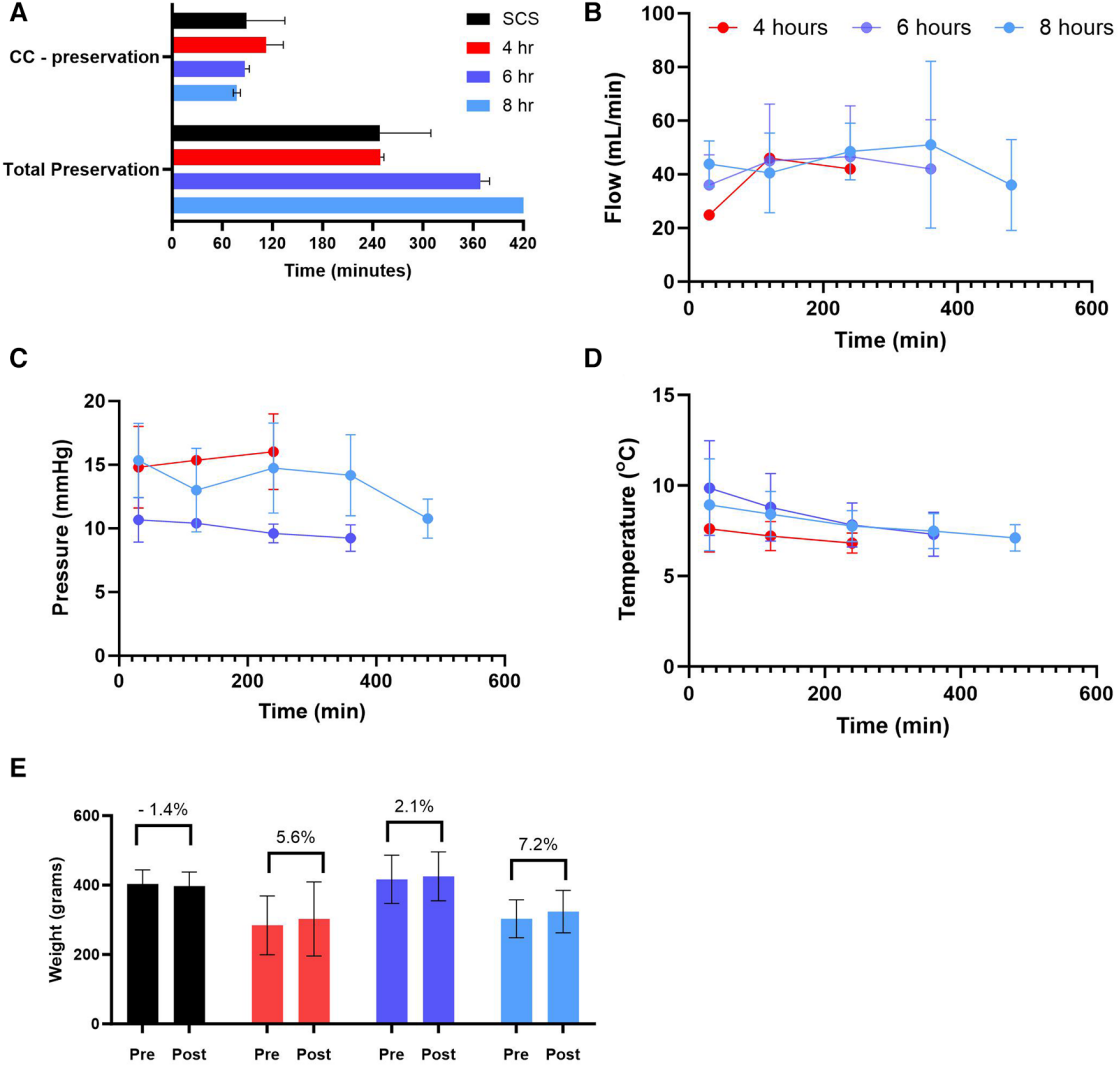
Preservation parameters during static cold storage (SCS) and hypothermic machine perfusion (HMP). (**A**) time (5) of cross-clamp (CC) to the start of preservation and total preservation time, (**B**) coronary flow during HMP, (**C**) perfusion pressure during HMP, (**D**) perfusion temperature during HMP, and (**E**) weight gain calculated post preservation for each group. Data expressed as means ± SEM.

Human donor hearts were offered for research by the local procurement organization within a very short distance. We calculated both the time it took from the cross-clamp (CC) to start preservation as well as the total preservation duration (Figure 3A). Our data show no significant difference in cc—preservation time among different groups as well as no profound differences in perfusion parameters. The average coronary flow for all groups combined was 41.86 ± 5.09 ml/min with an average flow not exceeding 50 mL/min for each group individually. The average perfusion pressure recorded 12.61 ± 4.04 mmHg, and the average temperature was 7.99 ± 1.5°C (Figures 3B-D). Thus, every heart was assessed for edema by weighing the heart prior to and post-preservation. Our results show no significant change in weight gain (%) following any preservation method (See Figure 3E).

### Biochemical markers of myocardial injury: lactates, CK-MB, and cardiac troponin I

**Figure 4:**
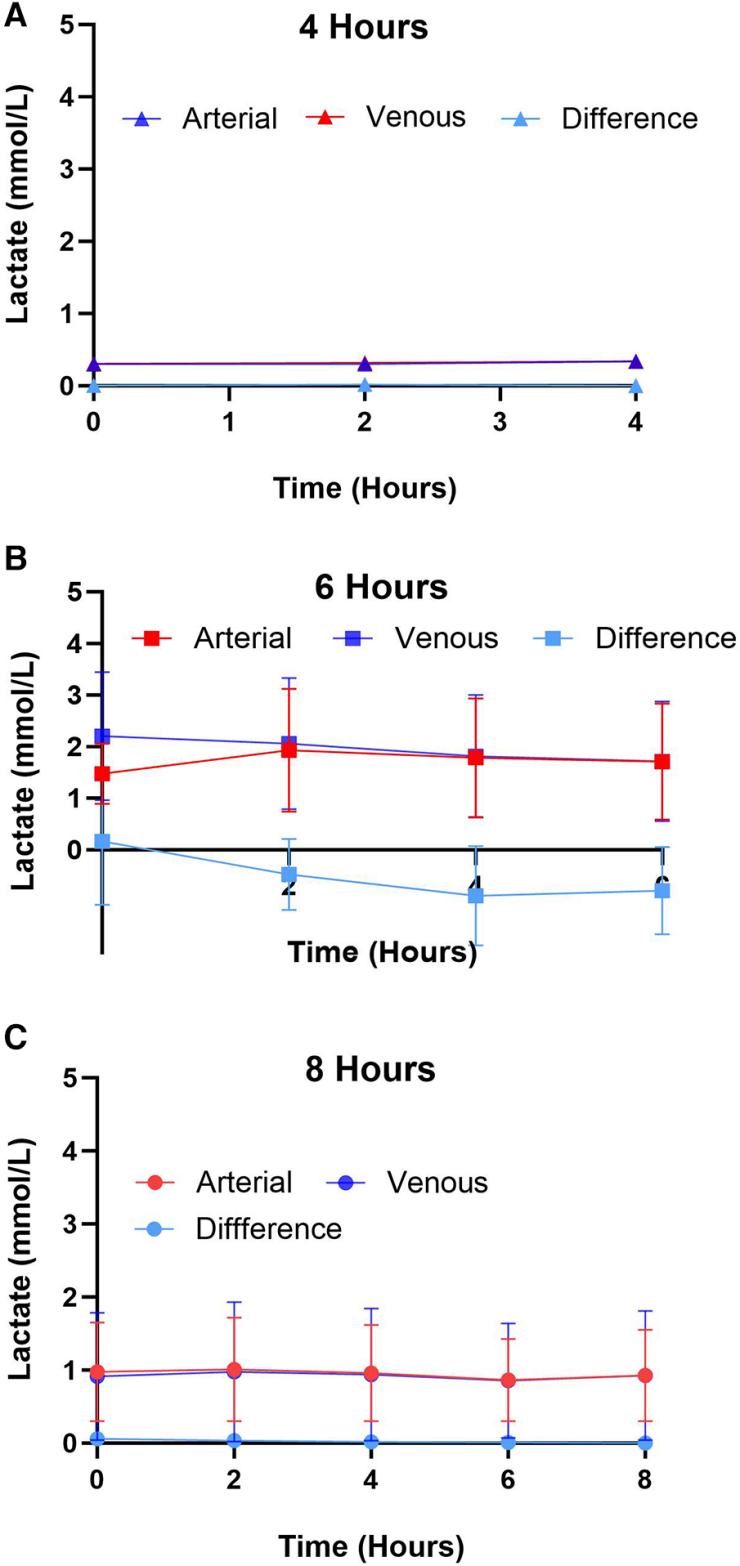
Arterial and venous lactate expression for different preservation groups including (**A**) 4 h, (**B**) 6 h, and (**C**) 8 h during hypothermic preservation. The difference in lactate expression, calculated as the arterial lactate concentration minus the venous lactate concentration, is also depicted on the graph. This provides insights into the net production or consumption of lactate by the heart during hypothermic perfusion. Samples were collected every two hours, and data displayed as means ± Std.

**Figure 5:**
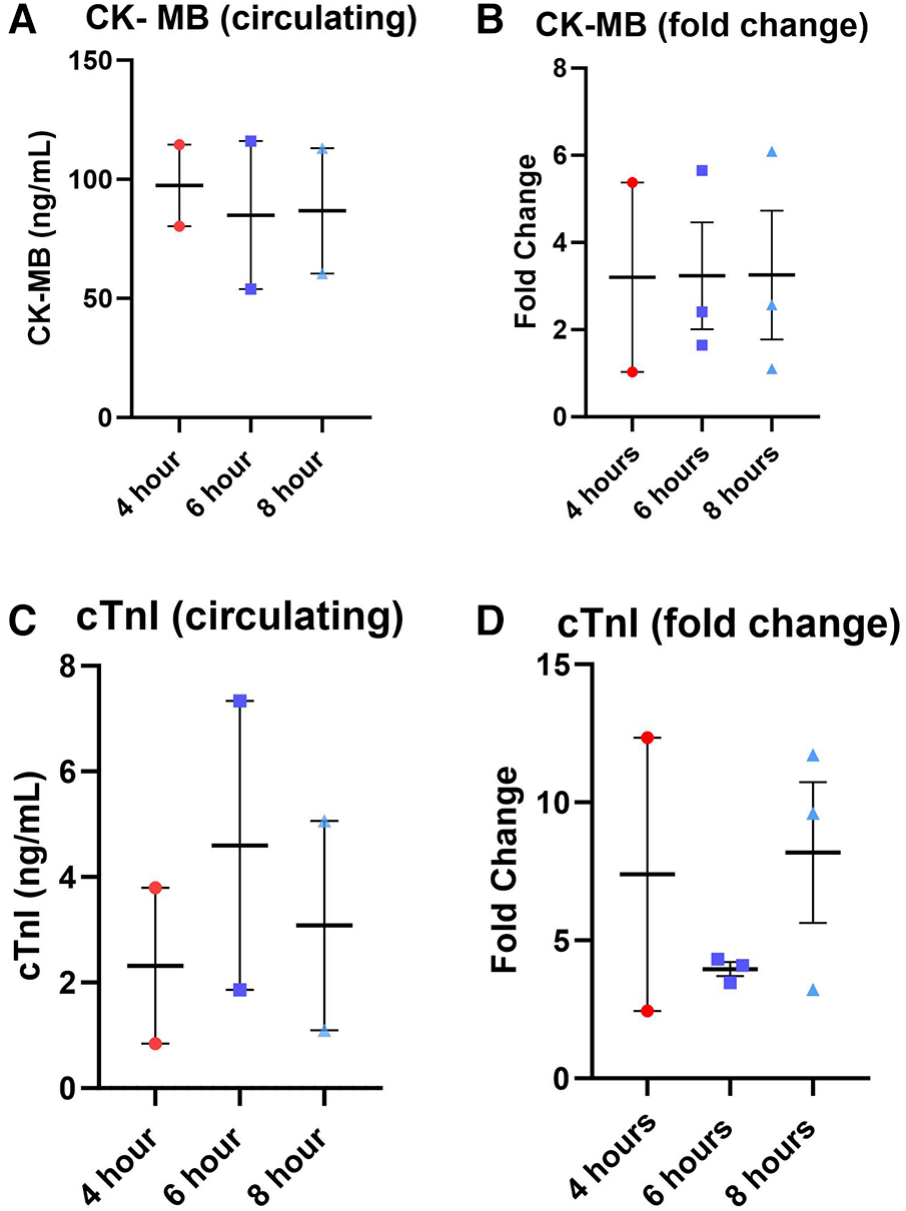
Total expression and fold change of circulating markers creatine kinase MB (CK-MB) and cardiac troponin I (cTnI) across all perfusion groups. (**A**) total expression of CK-MB followed by (**B**) the fold change, total expression of cTnI (**C**), and (**D**) the fold change. Values were obtained from venous samples at the beginning and at the end of perfusion. The fold change is calculated by dividing the expression level at the end by the baseline expression level. Each point represents an individual heart with indicators for the mean, lowest, and highest values. Data expressed as means ± SEM.

Lactate was measured from the coronary sinus and arterial samples to determine the tissue anaerobic state. Study results show lactate expression not exceeding 2 mmol/L across all groups (Figure 4). The lowest expression was detected in the 4-hour perfusion group with arterial lactate measured at 0.31 ± 0.02 and venous 0.32 ± 0.02 mmol/L. The 6-hour perfusion group resulted in arterial lactate concentration of 1.72 ± 0.19, while venous expression was 1.95 ± 0.22 mmol/L, and the 8-hour group had arterial lactate expression of 0.95 ± 0.06, while venous had 0.92 ± 0.04 mmol/L indicating a shift from lactate production to lactate consumption. Further, the negative lactate difference (venous—arterial) was observed in a 6-hour preservation group which may indicate a higher level of lactate clearance or lower lactate production in the venous circulation compared to the arterial circulation. This could be indicative of improved tissue perfusion and oxygenation, which are critical factors in the preservation of donor hearts. Next, we assessed the expression of one of the creatine phosphokinases (CPK) isoenzymes, creatine kinase myocardial band (CK-MB), which is commonly obtained after heart transplantation (HT) as an indicator of a myocardial injury of the donor heart (14) (Figures 5A–B). Another common marker for cardiac cell damage is cardiac troponins. The specific isoform I (cTnI) is commonly detected in heart transplant recipients as an indicator of graft failure (15). There was no significant difference detected of cTnI expression among different perfusion groups with 4-hour perfusion resulting in 2.32 ± 2.09 ng/mL, while 6 and 8-hour perfusion groups resulting in 4.60 ± 3.87 and 3.08 ± 2.81 ng/mL respectively (Figures 5C–D). Due to insufficient research on assessing CK-MB and cTnI expression in human hearts during HMP, drawing conclusions about the levels of these markers' is challenging. Yet, when focusing on fold change over total concentration, a consistent fold change of 3.2 was found in all groups for CK-MB expression. In contrast, the 6-hour preservation group exhibited the lowest fold change in cTnI expression at 3.96 ± 0.44.

### Myocardial oxygen consumption

**Figure 6:**
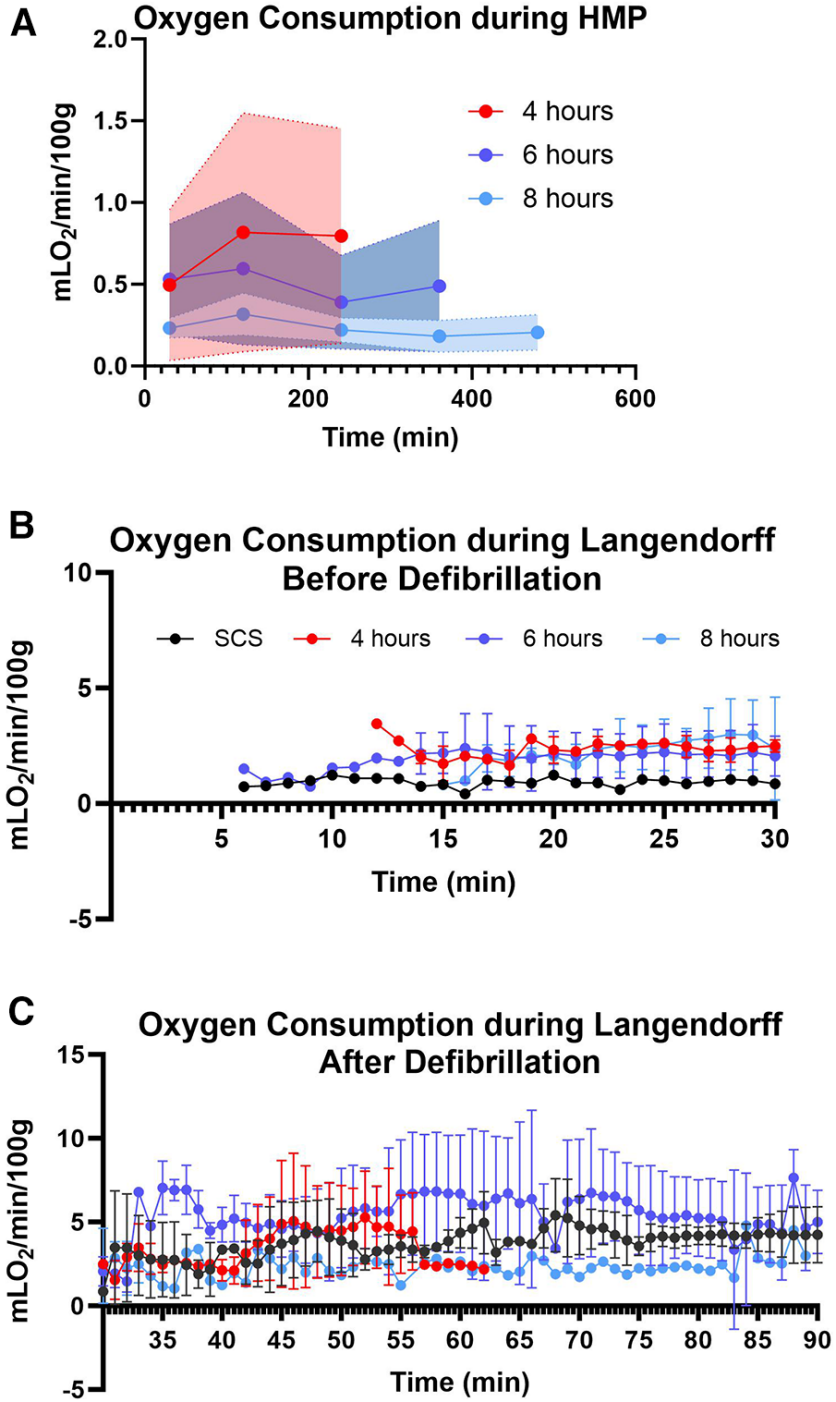
Myocardial oxygen consumption. (**A**) during hypothermic machine perfusion measured every two hours, (**B**) during reperfusion on the Landendorff system before defibrillation, and (**C**) on the Langendorff after defibrillation measured every 5 min. Shaded colors represent the error band area for each preservation group under the 95% confidence interval. Data displayed as means ± Std.

Myocardial oxygen consumption correlates with energy utilization from the cells and is considered a good marker to assess energy production and uptake by the myocardial tissue (16, 17). In this study, we assessed oxygen consumption during the perfusion and reperfusion phases (Figure 6). The average oxygen consumption for 4-hour preservation during HMP was 0.70 ± 0.1 mlO_2_/min/100 g, while for 6 and 8-hour preservation was 0.50 ± 0.09 and 0.23 ± 0.05 respectively. Hearts in different preservation groups show similar oxygen consumption trends which fall under the error band area representing the 95% confidence interval. Thus, our data is in alignment with findings from other investigators (18, 19). Oxygen consumption during the re-reperfusion stage shows normal oxygen consumption ranging from 2 to 5 mlO2/min/100 g across all groups (20).

### Assessment of cardiac function

**Figure 7:**
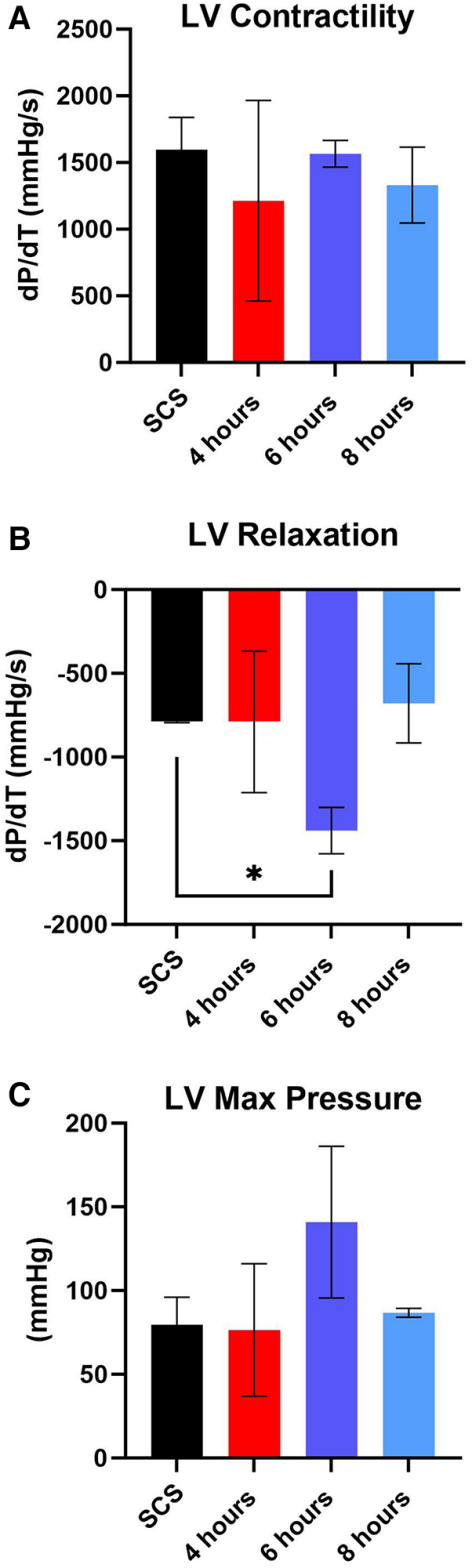
Assessment of left ventricular (LV) cardiac function during reperfusion. (**A**) LV contractility, (**B**) LV relaxation, and (**C**) LV maximum pressure. Data displayed as means ± SEM. Significance (*p* < 0.05) was determined by using the *t*-test.

After preservation, hearts were removed from the VP.S ENCORE® and placed in a Langendorff system for reperfusion and evaluation of cardiac contractility. This system assesses left ventricular dP/dT without preload and afterload, thereby allowing the evaluation of the intrinsic contractility of the left ventricle. No substantial change in left ventricle (3) contractility was detected among different groups with all of them corresponding to a normal range > 1,200 mmHg/s (Figure 7A) (21). However, there was a significant (*p* < 0.05) increase in LV relaxation in hearts perfused for 6 h (Figure 7B) and no significant difference in LV maximum pressure (Figure 7C).

### Myocardial histological assessment

**Figure 8:**
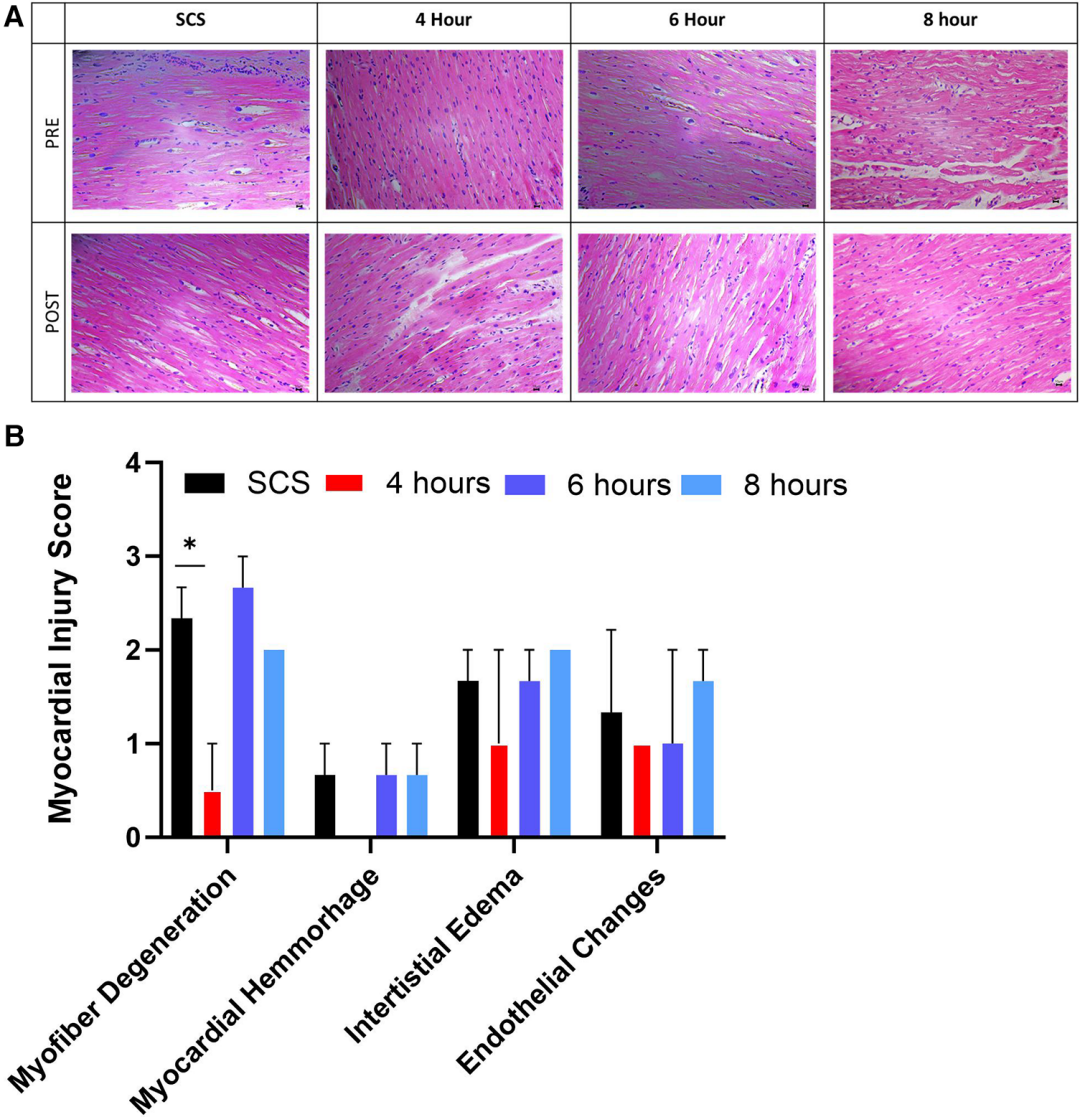
Myocardial histological assessment. (**A**) representative histological images from the apex area of hearts collected pre and post-preservation, followed by (**B**) myocardial injury score assigned by blinded pathologists. Myocardial injury score ranges from 0 to 3, where 0 is none, while 3 is severe. Data displayed as means ± Std. Significance (*p* < 0.05) was determined by using the *t*-test.

Representative histological images from hearts collected pre and post-preservation from the apex area showed no obvious ventricular differences within each heart (Figure 8A). Myocardial histological assessment post-preservation, characterized by the myocardial injury score, revealed a significant (*p* < 0.04) reduction in myofiber degeneration in the 4-hour preservation hearts group compared to the SCS group as characterized by the presence of leaky nuclei. There were no significant differences in other myocardial assessment parameters (myocardial hemorrhage, interstitial edema, and endothelial changes) between the standard of care and HMP groups.

### Gene expression analysis

**Figure 9:**
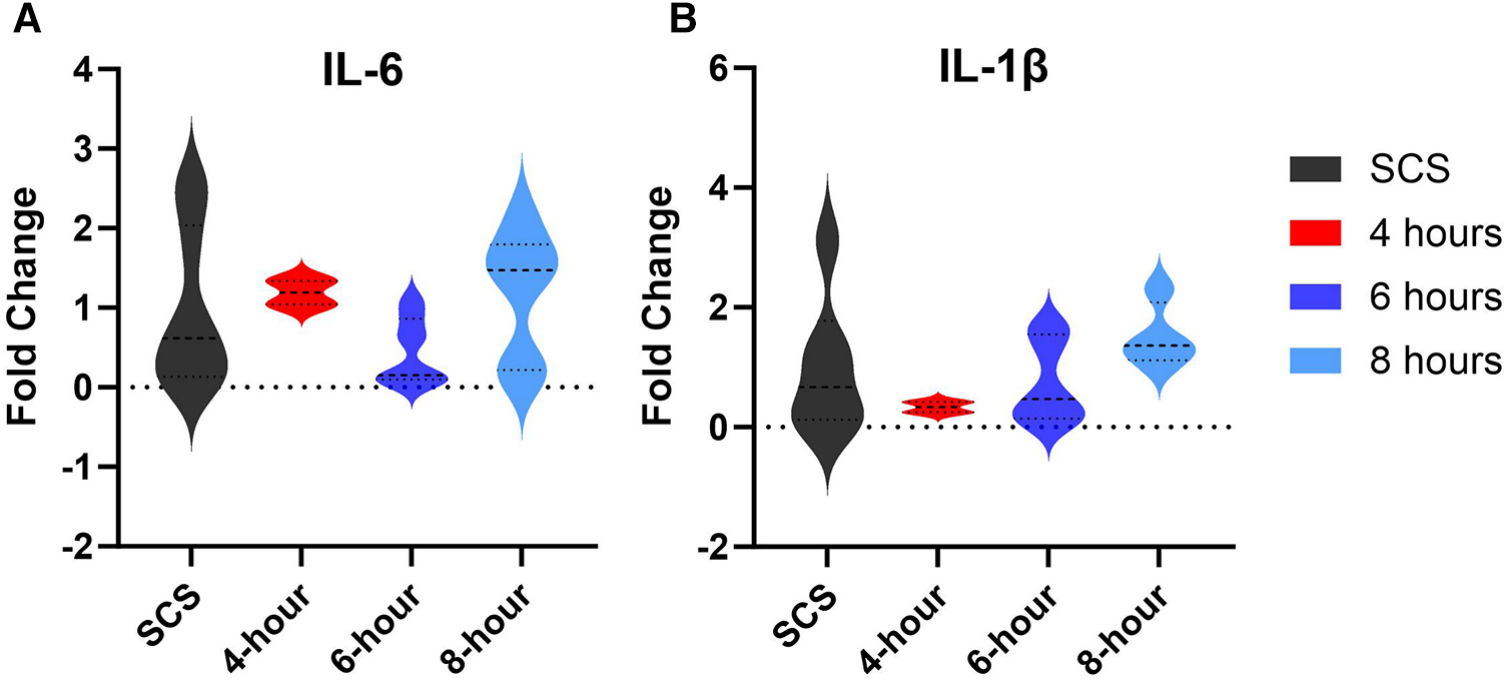
Gene expression of tissue inflammation markers. (**A**) IL-6 expression, (**B**) IL-1β expression. Tissues were obtained from the apex area after preservation and the fold change was calculated by the qRT-PCR. Data displayed as means ± Std and presented as violin plots with width representing the density of the data, and the shape showing the distribution of the data.

Gene expression analysis of inflammation markers interleukin (IL)-6 and interleukin (Il)-1β assessed in tissues collected at the end of each preservation method, showed no significant difference among different groups (Figure 9). However, some groups had profound downregulation of these inflammation markers, although not significant. As such, 6-hour perfusion led to a 45% downregulated expression of IL-6 and a 74% downregulation of IL-1β as visualized by the median dotted line in violin plots. Also, 4-hour perfusion led to a 34% downregulated expression of IL-1β. Elevated levels of these markers are known to be associated with inflammatory responses, cardiac remodeling, and heart failure (22, 23). However, their precise role in HMP and implications for clinical outcomes are yet to be delineated in future studies.

## Discussion

The growing demand for heart transplants remains a significant challenge, outpacing the availability of suitable organs, which is largely attributed to improved overall survival post-myocardial infarction and the aging global population (2). The availability of suitable hearts for transplantation continues as a major limit on the growing demand for heart transplantation. The primary component of this limitation is the 4-hour cross-clamp to cross-clamp preservation time imposed by the current standard of care, static cold storage. Each hour exceeding 4 h increases the probability of delayed graft function and primary graft failure leading to poorer morbidity and mortality (24, 25). As such, only hearts recovered within 4 h of the transplant site are typically accepted with a large proportion of suitable hearts being left unclaimed. Addressing this organ shortage is crucial, and machine perfusion technology, particularly when it is accessible and user-friendly, holds promise as a key strategy to enhance heart transplant utilization rates both in the United States and globally. The VP.S ENCORE®, a single use-device, weighing less than 23 Kg (50 pounds), and may be accompanied by only one technician with very minimal training (<30 min).

This study aimed to evaluate the feasibility of the VP.S ENCORE® device to preserve human donor-derived hearts not utilized for transplant for prolonged time. Study results revealed that hypothermic machine perfusion (HMP) between 4 and 10°C contributes to the reduction of the active metabolic state in cardiac grafts, as measured by low lactate and other myocardial markers expression, thereby minimizing the risk of the accumulation of toxic metabolites while maintaining cardiac viability (26). Examination of various metabolic markers, including oxygen consumption and lactate levels, demonstrated notable correlations with myocardial function during machine perfusion (4). The Arrhenius equation can be used to estimate the organ's oxygen demand during cardiac preservation. This equation is relevant to understanding the metabolic characteristics of human hearts preserved for a specific duration, and it provides insights into the rate of cellular processes and cardiac mitochondrial protection (27). Results indicate that the oxygen consumption during HMP was consistent with the Arrhenius prediction at 7°C for each of the groups and the results demonstrate that the VP.S ENCORE® delivered more than adequate levels of oxygen to the myocardium to cover its metabolic demand. Oxygen consumption prior to and post-defibrillation on the Langendorf was comparable in all groups and within the confidence interval predicted by the Arrhenius Equation at 37°C. Notably, study findings align with, and support results reported by other studies, reinforcing the consistency and significance of our observations. Additionally, the low perfusion pressures (<20 mmHg) minimized hydrostatic edema development to less than 8%. While the extent of the edema may not be the only factor in determining the suitability for transplantation, severe edema can impact the success of organ transplantation (28). One of the advantages of the VP.S ENCORE® device is no need for blood or blood-based products to be used. The solution of choice in this study was the Belzer MPS® UW Machine Perfusion solution, designed to keep the heart in diastolic arrest and to slow down cellular metabolism, reduce edema, and maintain cell integrity (17). While left ventricular contractility in the perfusion groups was similar to the SCS control, left ventricular relaxation following 6 h of perfusion was significantly greater. These findings are of particular significance in that cardiac function appears to have been maintained after an extended time of preservation. In addition, myocardial histological assessment and lower inflammation markers gene expression are consistent with the oxygen consumption and contractility data further reinforcing the notion that VP.S ENCORE® provides a superior preservation environment for cardiac grafts.

Despite these promising outcomes, it is essential to acknowledge the limitations of this study. Further testing and validation, particularly in larger cohorts and diverse clinical scenarios, are necessary to establish the generalizability and robustness of these findings. Additionally, the long-term effects of the VP.S ENCORE® device and its compatibility with various donor types warrant thorough exploration before widespread clinical application. The complexities and variability associated with human hearts, especially those procured from donation after circulatory death (DCD) donors, underscore the need for continued research and refinement of machine perfusion technologies. Preliminary data collected on using the VP.S ENCORE® to preserve DCD porcine hearts (data not shown) presents a promising avenue for the preservation of such hearts, potentially revolutionizing organ transplantation practices. Through controlled hypothermic perfusion and sophisticated monitoring capabilities, the VP.S ENCORE® device aims to mitigate ischemic injury, maintain metabolic homeostasis, and minimize the risk of organ rejection post-transplantation. Furthermore, the VP.S ENCORE® device may offer flexibility in transportation logistics and extend the window of opportunity for organ retrieval, enhancing accessibility to viable organs for transplantation. As ongoing research continues to explore its efficacy and refine its functionalities, the VP.S ENCORE® emerges as a promising tool in addressing the challenges associated with DCD heart preservation and advancing the field of organ transplantation.

The current study contributes valuable insights into the potential of the VP.S ENCORE® cardiac preservation device as a means to address the ongoing heart utilization challenges in heart transplantation. By building upon established principles of hypothermic machine perfusion and exploring innovative applications such as gene therapy and xenotransplantation, this research lays the groundwork for future advancements in organ preservation and transplantation strategies. While acknowledging the need for further investigation and refinement, the encouraging findings from this study emphasize the role of machine perfusion technology in shaping the future landscape of heart transplantation.

## Conclusions

The study's results demonstrate that human-derived donor hearts preserved in the VP.S ENCORE® for a prolonged time (4–8 h) had comparable and in most cases better outcomes than hearts stored using the standard of care preservation method. Given the hearts used in this study were rejected for transplant and had an initial lower ejection fraction (40%–50%) than typically accepted for transplantation, study findings suggest that the VP.S ENCORE® holds promise in preserving not only standard criteria but also “marginal” donor hearts, potentially further amplifying the utilization rates of heart transplants. Extending heart preservation time has the potential to improve organ allocation, increase transplant success rates, and enhance the overall efficiency of organ transplantation systems. However, careful consideration of associated challenges and risks is essential in implementing and optimizing these practices.

## Data Availability

The original contributions presented in the study are included in the article/Supplementary Materials, further inquiries can be directed to the corresponding author.
